# Cytotoxic Compounds from *Juglans sinensis* Dode Display Anti-Proliferative Activity by Inducing Apoptosis in Human Cancer Cells

**DOI:** 10.3390/molecules21010120

**Published:** 2016-01-21

**Authors:** Yoo Jin Lee, Jun Cui, Jun Lee, Ah-Reum Han, Eun Byul Lee, Ho Hee Jang, Eun Kyoung Seo

**Affiliations:** 1 College of Pharmacy, Graduate School of Pharmaceutical Sciences, Ewha Womans University, Seoul 120-750, Korea; yoojin0909@hanmail.net (Y.J.L.); andyhanandy@naver.com (A.-R.H.); 2 Department of Molecular Medicine, Graduate School of Medicine, Lee Gil Ya Cancer and Diabetes Institute, Gachon University, Incheon 406-840, Korea; cuijun928@naver.com (J.C.); jojnstar@naver.com (E.B.L.); 3 KM Convergence Research Division, Korea Institute of Oriental Medicine, Daejeon 34054, Korea; junlee@kiom.re.kr; 4 Korean Medicine Life Science, University of Science & Technology, Daejeon 34054, Korea; 5 Gachon Medical Research Institute, Gil Hospital, Incheon 405-760, Korea

**Keywords:** *Juglans sinensis* Dode, Juglandaceae, 8-hydroxy-2-methoxy-1,4-naphthoquinone, 5-hydroxy-2-methoxy-1,4-naphthoquinone, cytotoxicity, antiproliferative activity, apoptosis

## Abstract

Phytochemical investigation of the bark of *Juglans sinensis* Dode (Juglandaceae) led to the isolation of two active compounds, 8-hydroxy-2-methoxy-1,4-naphthoquinone (**1**) and 5-hydroxy-2-methoxy-1,4-naphthoquinone (**2**), together with 15 known compounds **3**–**17**. All compounds were isolated from this plant for the first time. The structures of **1** and **2** were elucidated by spectroscopic data analysis, including 1D and 2D NMR experiments. Compounds **1**–**17** were tested for their cytotoxicity against the A549 human lung cancer cell line; compounds **1** and **2** exhibited significant cytotoxicity and additionally had potent cytotoxicity against six human cancer cell lines, MCF7 (breast cancer), SNU423 (liver cancer), SH-SY5Y (neuroblastoma), HeLa (cervical cancer), HCT116 (colorectal cancer), and A549 (lung cancer). In particular, breast, colon, and lung cancer cells were more sensitive to the treatment using compound **1**. In addition, compounds **1** and **2** showed strong cytotoxic activity towards human breast cancer cells MCF7, HS578T, and T47D, but not towards MCF10A normal-like breast cells. They also inhibited the colony formation of MCF7, A549, and HCT116 cells in a dose-dependent manner. Flow cytometry analysis revealed that the percentage of apoptotic cells significantly increased in MCF7 cells upon the treatment with compounds **1** and **2**. The mechanism of cell death caused by compounds **1** and **2** may be attributed to the upregulation of Bax and downregulation of Bcl2. These findings suggest that compounds **1** and **2** may be regarded as potential therapeutic agents against cancer.

## 1. Introduction

*Juglans sinensis* Dode (Juglandaceae) is a deciduous tree indigenous to Eastern Asia and commonly known as the walnut tree. Previous phytochemical reports on this plant identified terpenoids, diarylheptanoids, naphthalenones, flavonoids, and phenolic compounds [1,2,3,4], which were related to its cytotoxic [1], neuroprotective [2], hepatic fibrosis inhibitory [3], and hepatoprotective [4] activities. The extracts of *J. sinensis* show antiasthma effects [5] and antioxidant activities on liver damage [6] and acute renal failure [7].

In previous reports on the anticancer effects of *Juglans* species, the extracts of root barks, fruits, or seeds of *J. regia* showed anti-proliferative activity against Caco-2 human colon cancer cells, HepG2 human liver cancer cells, and MDA-MB-231 human breast cancer cells [8,9,10]; the extract of seeds of *J. sinensis* protected UVB-induced human keratinocytes apoptosis [11]. Sesquiterpenes and triterpenes isolated from the leaves and twigs of *J. sinensis* inhibited the proliferation of immortalized rat hepatic stellate cells through apoptosis [1]; however, the mechanism of action of the anti-proliferation activity of the phenolic compounds of *J. sinensis* has not been investigated in detail.

Therefore, in continuation of our search for novel natural anticancer agents, we performed a bioactivity-guided fractionation to isolate and identify cytotoxic compound(s) from *J. sinensis*. Herein, we describe the separation and structure elucidation of such cytotoxic compounds, and furthermore, we evaluated their anti-proliferative and apoptotic activity to study mechanism of the cytotoxicity of these compounds in human cancer cells.

## 2. Results and Discussion

### 2.1. Phytochemical Characterization of the Bark of J. sinensis

**Figure 1:**
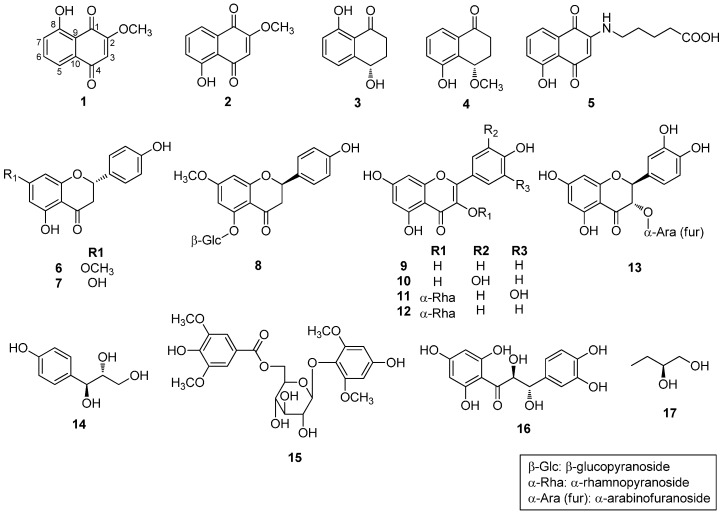
Chemical structures of the isolates **1**–**17** from the bark of *J. sinensis*.

**Figure 2:**
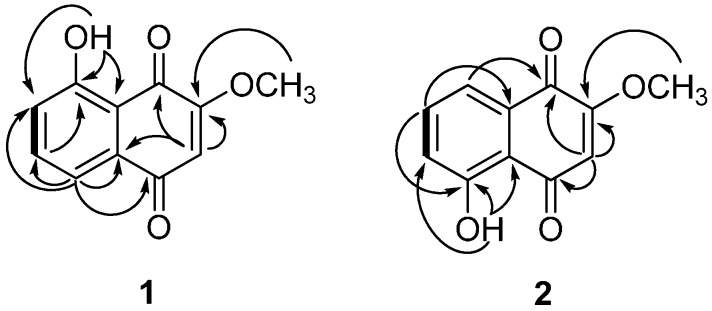
Key COSY (▬)and HMBC (→) correlations of **1** and **2**.

The structures of compounds **1** and **2** were identified by spectroscopic data interpretation (Figure 1). Compound **1** was obtained as a light brown powder. It gave a molecular ion peak at *m*/*z* 204.0421 [M]^+^ (calcd. for C_11_H_8_O_4_^+^, 204.0423) in HRESIMS, corresponding to an elemental formula of C_11_H_8_O_4._ The UV spectrum of **1** showed an absorption maximum at 263 nm, indicating the presence of an aromatic system. The ^1^H-NMR spectrum of **1** showed signals for a hydroxy group at δ_H_ 11.75 (1H, s), an aromatic ring system at δ_H_ 7.25 (1H, dd, *J* = 2.8, 6.4 Hz) and 7.63 (overlapped 2H, d, *J* = 2.8, 6.4 Hz), an aromatic singlet at δ_H_ 6.11 (1H, s), and a methoxy group at δ_H_ 3.92 (3H, s). The ^13^C-NMR spectrum of **1** showed signals for two carbonyls at δ_C_ 184.9 (C-1) and 183.9 (C-4), two oxygenated quaternary carbons at δ_C_ 162.0 (C-8) and 160.1 (C-2), four aromatic methines at δ_C_ 137.2 (C-6), 123.9 (C-7), 118.9 (C-5), and 110.5 (C-3), and two quaternary carbon signals at δ_C_ 132.1 (C-10) and 114.3 (C-9). These spectral data supported the notion that compound **1** contained a naphthalenedione, as evidenced by the HMBC correlations of H-3/C-1, C-2, C-10, H-5 and H-6 (overlapped peak)/C-4, C-6, C-7, C-9, C-10, H-7/C-6, C-9. The positions of the hydroxyl group at C-8 and the methoxy group at C-2 were confirmed by the HMBC correlations of OH/C-7, C-8, C-9 and OCH_3_/C-2, respectively (Figure 2). Based on these observations and by comparison of its spectral data with literature values [12,13], compound **1** was identified as 8-hydroxy-2-methoxy-1,4-naphthoquinone (Figure 1).

Compound **2** was obtained as a light brown powder and showed a molecular ion peak at *m*/*z* 204.0421 [M]^+^ (calcd. for C_11_H_8_O_4_^+^, 204.0423) in HRESIMS, corresponding to an elemental formula of C_11_H_8_O_4._ The ^1^H- and ^13^C-NMR spectra of **2** were similar to those of **1**, except for the signals of the aromatic ring system. The ^1^H-NMR spectrum of **2** showed an aromatic ring system at δ_H_ 7.28 (1H, dd, *J* = 1.2, 8.1 Hz), 7.59 (1H, t, *J* = 8.1 Hz), 7.68 (1H, dd, *J* = 1.2, 8.1 Hz). The positions of the hydroxyl group at C-5 and the methoxy group at C-2 were confirmed by the HMBC correlations of OH/C-5, C-6, C-10 and OCH_3_/C-2, respectively (Figure 2). Therefore, compound **2** was identified as 5-hydroxy-2-methoxy-1,4-naphthoquinone (Figure 1) by comparison of its spectral data with literature values [14].

The known compounds identified in the present investigation are as follows: (4*S*)-isosclerone (**3**) [15,16], (4*S*)-5-hydroxy-4-methoxy-α-tetralone (**4**) [16], juglonbutine (**5**) [17], (2*S*)-sakuranetin (**6**) [18,19,20], (2*S*)-naringenin (**7**) [19,20,21], (2*R*)-sakuranin (**8**) [19,20,22], kaempferol (**9**) [22], quercetin (**10**) [23], quercitrin (**11**) [24], afzelin (**12**) [24], (–)-taxifolin 3-*O*-α-l-arabinofuranoside (**13**) [25], (*erythro*)-1-(4-hydroxyphenyl)-1,2,3-propanetriol (**14**) [26], 4′-hydroxy-2′,6′-dimethoxyphenol 1-*O*-β-d-(6-*O*-syringoyl) glucopyranoside (**15**) [27], (7*S*,8*S*)-cilicione b (**16**) [28], and (2*R*)-1,2-butanediol (**17**) [29] by comparison of their physical and spectroscopic data with the literature data (Figure 1). These compounds **3**–**17** were all isolated as the constituents of this plant for the first time. Moreover, **14** and **16** have not been found in the family Juglandaceae.

### 2.2. Biological Evaluations of Compounds

#### 2.2.1. Identification of Cytotoxic Compounds

The cytotoxic activities of the MeOH extract, solvent-partitioned fractions, and the compounds isolated from *J. sinensis* were examined on the A549 human non-small cell lung cancer cell line at various concentrations for 24 h. Inhibitory concentration (IC_50_) values were calculated from their cell viability curves. Because the MeOH extract showed cytotoxic activity against A549 cells, this extract was partitioned into hexane, ethyl acetate, butanol, and aqueous soluble fractions. As shown in Table 1, the ethyl acetate fraction was the major fraction responsible for the cytotoxic activity compared to other fractions.

Individual compounds were isolated from the ethyl acetate fraction, and their IC_50_ values were determined against A549 cells. The cells were treated with 0–50 μM of compounds **1** to **17** for 24 h. Compounds **1** and **2** showed strong cytotoxicity against A549 cells, with the IC_50_ values of 1.82 and 1.33 μM, respectively, whereas other compounds were inactive (IC_50_ > 10 μM, Table 1). Based on the cytotoxic potency and selectivity, compounds **1** and **2** were selected as the potential anticancer compounds and for further investigation of their cytotoxicity against different human cancer cell lines.

**Table 1:** Cytotoxicity of the extract, fractions, and compounds isolated from *J. sinensis.*

Samples	IC_50_
Methanol extract	153.4 ± 10.61 ^a^
Hexane fraction	41.92 ± 3.68 ^a^
Ethyl acetate fraction	31.23 ± 0.67 ^a^
Butanol fraction	61.51 ± 2.00 ^a^
Aqueous fraction	155.3 ± 10.71 ^a^
**1**	1.33 ^b^
**2**	1.82 ^b^
**3**–**17**	>10 ^b^

^a^ range of activity in μg/mL; ^b^ range of activity in μM.

#### 2.2.2. Cytotoxic Effects of Compounds **1** and **2** against Various Human Cancer Cells

**Figure 3:**
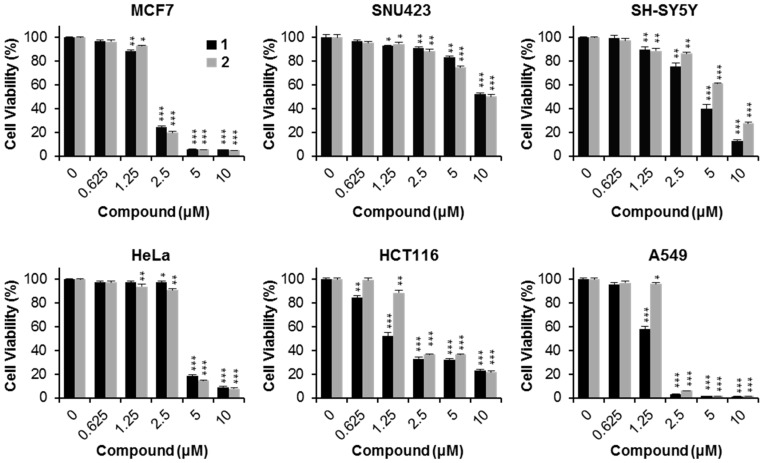
Cytotoxicity of compounds **1** and **2** on human cancer cell lines. Cells were treated with compounds **1** and **2** at the indicated concentration for 24 h. Cytotoxicity was evaluated by the cell viability assay. * *p* < 0.05; ** *p* < 0.01; *** *p* < 0.001, compared to the control.

**Figure 4:**
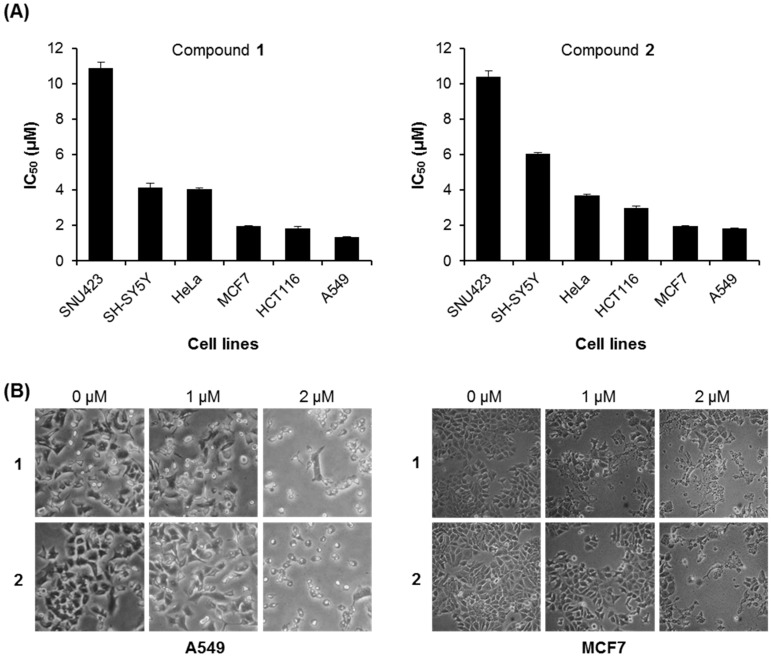
IC_50_ values of compounds **1** and **2** against human cancer cell lines. (**A**) The IC_50_ values of compounds **1** and **2** for indicated cell lines; (**B**) Cell morphology of A549 and MCF7 cells treated with compounds **1** and **2** for 24 h under microscopic observation (400×).

Different human cancer cell lines were treated with compounds **1** and **2** using serial dilution concentrations (10, 5, 2.5, 1.25, 0.625, and 0 μM). As shown in Figure 3, the cell viability rates decreased with increasing concentrations of compounds **1** and **2** in a dose-dependent manner. Compound **1** showed significant cytotoxic activity for all the cancer cells tested (MCF7, SNU423, SH-SY5Y, HeLa, HCT116, and A549), with IC_50_ values of 1.95 ± 0.05, 10.87 ± 0.33, 4.13 ± 0.27, 4.07 ± 0.06, 1.83 ± 0.12, and 1.33 ± 0.02 μM, respectively (Table 2 and Figure 4A). Similarly, compound **2** demonstrated potent cytotoxicity against all six cancer cells (MCF7, SNU423, SH-SY5Y, HeLa, HCT116, and A549), with IC_50_ values of 1.96 ± 0.04, 10.42 ± 0.31, 6.07 ± 0.05, 3.70 ± 0.05, 3.00 ± 0.12, and 1.82 ± 0.03 μM, respectively (Table 2 and Figure 4A). Especially, compound **1** displayed strong activity against MCF7 breast cancer, HCT116 colon cancer, and A549 lung cancer cells. A549 and MCF7 cells were used to further evaluate the cytotoxic effect of compounds **1** and **2**. In addition, when compounds **1** or **2** were treated in A549 and MCF7 cells for 24 h, the cell morphology became more round and floated compared to the untreated healthy cells, showing a dissimilar cytoskeleton (Figure 4B).

**Table 2:** IC_50_ values (μM) of compounds **1** and **2** against human cancer cell lines.

Compounds	MCF7	SNU423	SH-SY5Y	HeLa	HCT116	A549
**1**	1.95 ± 0.05	10.87 ± 0.33	4.13 ± 0.27	4.07 ± 0.06	1.83 ± 0.12	1.33 ± 0.02
**2**	1.96 ± 0.04	10.42 ± 0.31	6.07 ± 0.05	3.70 ± 0.05	3.00 ± 0.12	1.82 ± 0.03

The data are expressed as mean ± SD of three independent experiments.

#### 2.2.3. Cancer Cell Specific Cytotoxicity of Compounds **1** and **2**

**Figure 5:**
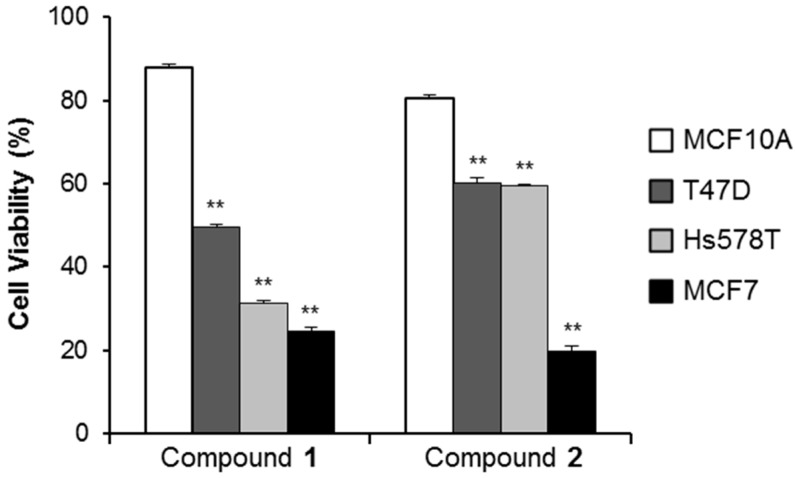
Cytotoxic effects in the normal and breast cancer cell lines. Cells were exposed to 2.5 μM of compounds **1** and **2** for 24 h. ** *p* < 0.01, compared to the MCF10A cells. Data presented are mean ± SD from three independent observations.

To examine whether compounds **1** and **2** inducesd cancer cell specific cytotoxicity, human normal mammary epithelial cells (MCF10A) and breast cancer cell lines (MCF7, Hs578T, T47D) were treated with 2.5 μM of compounds **1** and **2** for 24 h. As shown in Figure 5, the cell viability of compound **1**- or **2**-treated MCF10A cells was 87.83% ± 0.94% and 80.45% ± 0.69%, respectively. However, the cytotoxic effect of compounds **1** and **2** was higher in the breast cancer cells than in normal-like breast cells. Cell viability of T47D, Hs578T and MCF7 treated with compound **1** was 49.50% ± 0.76%, 31.40% ± 0.56%, and 24.54% ± 1.08%, respectively. Cell viability of T47D, Hs578T, and MCF7 cells treated with compound **2** was 60.29% ± 1.21%, 59.39% ± 0.31%, and 19.89% ± 1.10%, respectively. These findings indicate that compounds **1** and **2** displayed significant cytotoxicity to human breast cancer cells.

#### 2.2.4. Anti-Proliferative Activity of Compounds **1** and **2**

**Figure 6:**
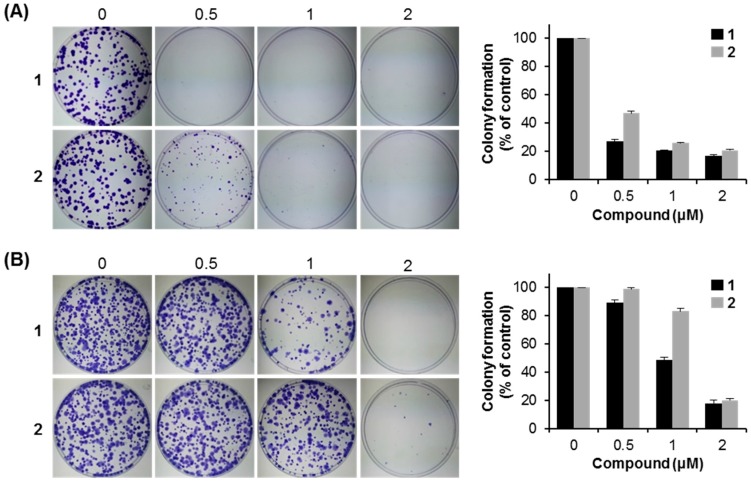

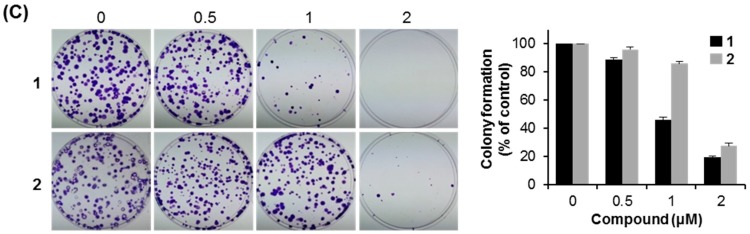
Inhibitory effects of compounds **1** and **2** on colony formation capacity of human cancer cells. The colony formation assays of MCF7 (**A**); A549 (**B**); and HCT116 cells (**C**) treated with compounds **1** and **2** at the indicated concentrations for two weeks. Data presented are mean ± SD from three independent observations.

To explore the anticancer properties of compounds **1** and **2**, colony formation assays were performed. MCF7 cells were incubated with compounds **1** and **2** at various concentrations (0, 0.5, 1, and 2 μM) for two weeks. Compounds **1** and **2** suppressed the colony formation of MCF7 breast cancer cells in a dose-dependent manner, and compound **1** was more sensitive than compound **2** for MCF7 cells (Figure 6A). To further confirm these results, the inhibitory effect of colony formation was also examined using another two cell lines, HCT116 colon cancer cells and A549 lung cancer cells, at the same concentrations of compounds **1** and **2** (Figure 6B,C). The results show that compounds **1** and **2** can inhibit the colony formation capacity; especially compound **1** was also more sensitive than compound **2** for both A549 and HCT116 cells, which was consistent with that of MCF7 cells. Taken together, compounds **1** and **2** showed the inhibitory effect of the colony formation in human cancer cells, and compound **1** was more sensitive than compound **2** for human cancer cells.

#### 2.2.5. Apoptotic Activity of Compounds **1** and **2**

**Figure 7:**
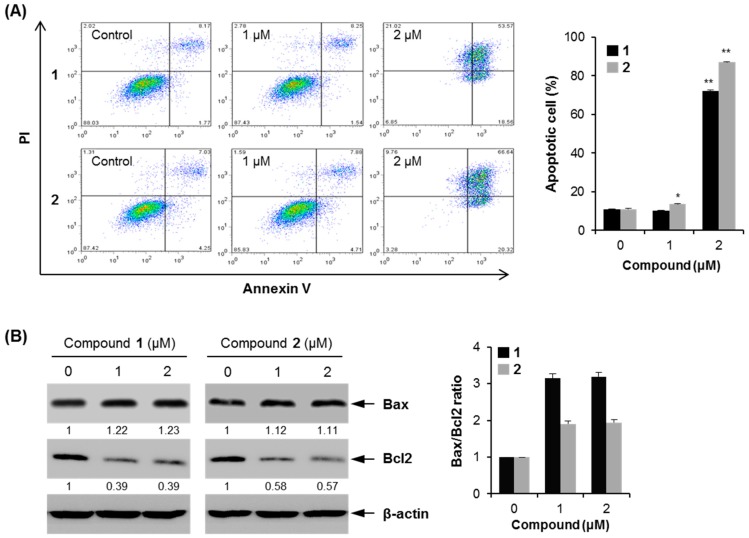
Cellular apoptosis by compounds **1** and **2** in MCF7 cells. (**A**) Flow cytometry was performed to measure cellular apoptosis. * *p* < 0.05; ** *p* < 0.01 *vs.* control; (**B**) western blot was performed using the indicated antibodies. The numbers represent the mean fold change of the respective protein levels in compounds-treated cells relative to the control cells. The Bax/Bcl2 ratio is shown as fold changes compared to that of the untreated cells.

To determine whether compounds **1** and **2** caused apoptosis, flow cytometry was performed using Annexin V-FITC and propidium iodide (PI) double staining assay in MCF7 cells. At 24 h after treatment with **1** and **2**, the proportion of apoptotic cells was 61% higher in 2 μM compound **1**-treated MCF7 cells than in the control cells (72.19% ± 0.36% *vs.* 10.79% ± 0.19%, *p* < 0.01) and 76% higher in 2 μM compound **2**-treated MCF7 cells than in the control cells (87.02% ± 0.18% *vs.* 11.04% ± 0.26%, *p* < 0.01) (Figure 7A). 

Moreover, compound **1** induced cell necrosis at 2 μM compared to the untreated control group (2.02% ± 0.62% *vs.* 21.02% ± 1.12%); however, compound **2** did not induce cell necrosis (Figure 7A). These findings indicate that compounds **1** and **2** could induce apoptosis and inhibit cancer cell growth. To study the mechanism of compounds **1** and **2**-induced apoptosis, the expression levels of apoptosis-related proteins were measured using western blotting (Figure 7B). The treatment with these compounds increased the expression level of Bax, a pro-apoptotic protein, whereas the expression level of Bcl2, an anti-apoptotic protein, decreased by the treatment with these compounds. When the expression level of Bax was represented as ratios to the level of Bcl2, the treatment with these compounds increased the ratio of Bax/Bcl2, and especially a significant elevation in the ratio of Bax/Bcl2 in compound **1**-treated cells was observed. Altogether, these findings demonstrate that compounds **1** and **2** induced apoptosis by regulating pro- and anti-apoptotic genes.

### 2.3. Discussion

Breast cancer is the second most common reason for death in females worldwide [30]. In this study, 17 phenolic compounds **1**–1**7** were isolated from the bark of *J. sinensis*; their cytotoxic activities were tested against diverse human cancer cells. Among them, compounds **1** and **2** exhibited cytotoxic activities and inhibited human cancer cell growth, which was in agreement with the previous reporters for similar compounds [31,32,33].

2-Methoxy-1,4-naphthoquinone (MNQ) exerts anticancer activity by the induction of apoptosis [34]. Our data demonstrate that compounds **1** and **2** induced apoptotic characteristics such as cytoplasm retraction, bleb formation, and the condensation of nuclear material [35,36], in a dose-dependent manner (Figure 4B). Identified apoptotic pathways in cells can divide into two pathways mediated by: (i) the death receptor and (ii) mitochondria [37,38]. Recent report showed that MNQ promotes cancer cell death by a reactive oxygen species (ROS)-dependent mechanism [34]. Since compounds **1** and **2** have the same skeleton as MNQ, compounds **1** and **2** should have similar activity as MNQ based on their structures. The mitochondrial-mediated apoptosis is regulated by the Bcl2 protein family [39]. Pro-apoptotic protein Bax transposes to the mitochondrial outer membrane, and anti-apoptotic protein Bcl2 expression decreases followed by cytochrome c release inducing cell apoptosis. Our results show that compounds **1** and **2** induced cell apoptosis by reducing Bcl2 protein and increasing Bax protein, therefore they may induce cell apoptosis by a mitochondrial-mediated pathway.

## 3. Experimental Section

### 3.1. General Procedures

Optical rotations were measured on a P-1010 polarimeter (Jasco, Tokyo, Japan). UV spectra were recorded on a U-3000 spectrophotometer (Hitachi, Tokyo, Japan). CD spectra were obtained using a J-810 CD-ORD spectropolarimeter (Jasco). HR-ESI mass spectrometric analyses were performed with an ACQUITY UPLC system (Waters Co., Milford, MA, USA) coupled to a Micromass Q-TOF Micromass spectrometer and a 6220 Accurate-Mass TOF LC/MS system (Agilent Technologies, Inc., Santa Clara, CA, USA). The 1D and 2D NMR experiments were performed on a Unity Inova 400 MHz FT-NMR instrument (Varian, Inc., Palo Alto, CA, USA) with tetramethylsilane (TMS) as an internal standard. Thin-layer chromatographic (TLC) analysis was performed on Kieselgel 60 F_254_ (Merck, Darmstadt, Germany), with visualization under UV light (254 and 365 nm) and 10% (*v*/*v*) sulfuric acid spray followed by heating (120 °C, 5 min). Silica gel (230–400 mesh, Merck), YMC Gel ODS-A (12 nm, S-150 μm; YMC Co., Kyoto, Japan), and Sephadex LH-20 (Pharmacia Co., Uppsala, Sweden) were used for column chromatography (CC). (Please provide us the full information of company, city, country for all the equipment). 

### 3.2. Materials and Chemicals

The barks of *J. sinensis* were collected at the Medicinal Plant Garden, College of Pharmacy, Ewha Womans University, in August 2010, and identified by Prof. Je-Hyun Lee (Dongguk University, Geongju 780-714, Korea). A voucher specimen (No. EA310) was deposited at the Natural Product Chemistry Laboratory, College of Pharmacy, Ewha Womans University. Cell culture reagents were purchased from WelGENE (Daegu, Korea). The EZ-CyTox Cell Viability assay kit was purchased from Daeil Lab Service Co. (Seoul, Korea). Formaldehyde, crystal violet and DMSO were obtained from Sigma (St. Louis, MO, USA). The EzWay Annexin V-FITC apoptosis detection kit was purchased from KOMA Biotech (Seoul, Korea). Xpert protease inhibitor cocktail was purchased from GenDEPOT (Barker, TX, USA). Primary antibodies against Bcl2, Bax and β-actin were purchased by Santa Cruz Biotechnology (Santa Cruz, CA, USA), and horseradish peroxidase (HRP)-conjugated secondary antibody was obtained from Jackson Immuno Research laboratories, Inc. (West Grove, PA, USA). The enhanced chemiluminescence (ECL) kit was obtained from Advansta Inc. (Advansta, CA, USA).

### 3.3. Extraction and Isolation

The air-dried barks of *J. sinensis* (18 kg) were extracted with MeOH (25 L × 4) for 24 h at room temperature. The solvent was evaporated *in* *vacuo* to provide a concentrated MeOH extract (1.6 kg), which was then diluted with distilled water (1.2 L) to afford an aqueous methanolic solution. The aqueous solution was sequentially partitioned with *n*-hexane (3 L × 3), EtOAc (2 L × 3), and *n*-BuOH (3 L × 3) to afford the following fractions: *n*-hexane (170 g), EtOAc (755 g), and *n*-BuOH-soluble (414 g) fractions. The partial EtOAc fraction (350 g) was subjected to silica gel column chromatography (CC) (CHCl_3_–MeOH, 99:1 to 2:1, *v*/*v*) to yield ten fractions (E01–E10). Fraction E09 (66.84 g) was recrystallized using MeOH to afford **11** (10 g). Fraction E02 (6.5 g) was applied to silica gel CC (CHCl_3_–acetone, 99.5:0.5 to 98:2, *v*/*v*), leading to fifteen sub-fractions (E0201–E0215). Among them, sub-fraction E0205 (99.5:0.5, 50 mg), E0206 (99.5:0.5, 45 mg), and E0214 (98.5:1.5, 598.78 mg) were identified as **1** (40 mg), **2** (50 mg), and **6** (500 mg), respectively. Sub-fraction E0215 (2.3 g) was subjected to silica gel CC (CHCl_3_–acetone, 99:1 to 98:2, *v*/*v*) and was further purified by RP-C_18_ CC (MeOH–water, 1:1 to 2:1, *v*/*v*) to afford **3** (7 mg). Fraction E0210 (166 mg) was purified by RP-C_18_ CC (CH_3_CN–water, 1:9 to 1:1, *v*/*v*) to furnish **4** (4 mg). Fraction E07 (56 g) was applied to silica gel CC (CHCl_3_–MeOH, 99:1 to 1:1, *v*/*v*), leading to twelve sub-fractions (E0701–F0712). Sub-fraction E0711 (7.6 g) was subjected to RP-C_18_ CC (MeOH–water, 1:3 to 1:1, *v*/*v*) and Sephadex LH-20 CC (100% MeOH), affording **5** (8 mg), **13** (7 mg), and **17** (2 mg). The combined sub-fractions, E0707 and E0708 (17 g), were further purified by silica gel CC (CHCl_3_–acetone, 99:1 to 1:1, *v*/*v*) to afford **9** (116 mg), **10** (8 mg), and **16** (70 mg). Sub-fraction E0710 (11 g) was subjected to silica gel CC (CHCl_3_–MeOH, 99:1 to 1:1, *v*/*v*) to obtain **12** (9 mg). The combined fractions, E04 and E05 (4.6 g) was applied to silica gel CC (CHCl_3_–acetone, 99:1 to 1:1, *v*/*v*) leading to 15 sub-fractions (E0401–F0415) and **7** (36 mg). Sub-fraction E0409 (161 mg) was further purified by RP-C_18_ CC (MeOH–water, 1:1 to 3:1, *v*/*v*), affording **14** (2 mg). The BuOH fraction (414 g) was subjected to silica gel CC (CH_2_Cl_2_–MeOH, 98:2 to 1:1, *v*/*v*) to yield 13 fractions (B01–B13). The combined sub-fractions, B06, B07, and B08 (6.2 g), were applied to RP-C_18_ CC (MeOH–water, 1:4 to 1:2, *v*/*v*) providing eighteen sub-fractions (B0601–B0618) and **8** (70 mg). The combined sub-fractions, B0609 and B0610 (811 mg), were purified by silica gel CC (CH_2_Cl_2_–MeOH, 49:1 to 1:1, *v*/*v*) to obtain **15** (100 mg).

*8-Hydroxy-2-methoxy-1,4-naphthoquinone* (**1**). Light brown needle-like crystals. ^1^H-NMR (CDCl_3_, 400 MHz) δ 11.75 (1H, s, OH), 7.63 (2H, dd, *J* = 2.8 , 6.4 Hz, overlapped H-5 and H-6), 7.25 (1H, dd, *J* = 2.8, 6.4 Hz, H-7), 6.16 (1H, s, H-3), 3.92 (3H, s, OCH_3_); ^1^H-NMR (DMSO, 400 MHz) δ 11.57 (1H, s, OH), 7.74 (1H, t, *J* = 7.7 Hz, H-6), 7.50 (1H, dd, *J* = 7.7, 1.0 Hz, H-5), 7.25 (1H, dd, *J* = 7.7, 1.0 Hz, H-7), 6.34 (1H, s, H-3), 3.87 (3H, s, OCH_3_); ^13^C-NMR (CDCl_3_) δ 184.9 (C-1), 183.9 (C-4), 162.0 (C-8), 160.1 (C-2), 137.2 (C-6), 132.1 (C-10), 123.9 (C-7), 118.9 (C-5), 114.3 (C-9), 110.5 (C-3), 56.6 (OCH_3_); HRESIMS *m*/*z* 204.0421 [M]^+^ (calcd. for C_11_H_8_O_4_^+^, 204.0423).

*5-Hydroxy-2-methoxy-1,4-naphthoquinone* (**2**). Light brown powder. ^1^H-NMR (CDCl_3_, 400 MHz) δ 12.23 (1H, s, OH), 7.68 (1H, dd, *J* = 1.2, 8.1 Hz, H-8), 7.59 (1H, t, *J* = 8.1 Hz, H-7), 7.28 (1H, dd, *J* = 1.2, 8.1 Hz, H-6), 6.11 (1H, s, H-3), 3.93 (3H, s, OCH_3_); ^13^C-NMR (CDCl_3_) δ 190.8 (C-4), 179.4 (C-1), 161.1 (C-2), 161.1 (C-5), 135.5 (C-7), 131.1 (C-9), 125.2 (C-6), 119.6 (C-8), 114.2 (C-10), 109.5 (C-3), 56.6 (OCH_3_); HRESIMS *m*/*z* 204.0421 [M]^+^ (calcd. for C_11_H_8_O_4_^+^, 204.0423).

*(4S)-Isosclerone* (**3**). Amorphous solid. CD (MeOH, *c* = 5.62 × 10^−3^ M) ∆ε (nm): −27.6 (255), +10.7 (203) [16].

*(4S)-5-Hydroxy-4-methoxy-α-tetralone* (**4**). Amorphous powder. CD (MeOH, *c* = 5.20 × 10^−3^ M) ∆ε (nm): −0.8 (345), +10.8 (230) [16].

*(2S)-Sakuranetin* (**6**). Pale yellow powder. CD (MeOH, *c* = 3.5 × 10^−3^ M) +1.5 (334), −23.6 (293) [19,20].

*(2S)-Naringenin* (**7**). Pale brown powder. CD (MeOH, *c* = 3.7 × 10^−3^ M) +2.0 (331), −21.6 (291) [19,20].

*(2R)-Sakuranin* (**8**). White amorphous powder. CD (MeOH, *c* = 3.3 × 10^−3^ M) −1.9 (333), +9.2 (290) [19,20].

*(–)-Taxifolin 3-O-α-l-arabinofuranoside* (**13**). Amorphous solid. ${\left[\alpha \right]}_{D}^{24}$ −26.7 (*c* 0.1, MeOH) [40].

(*erythro*)-1*-(4-Hydroxyphenyl)-1,2,3-propanetriol* (**14**). Brown needle. ${\left[\alpha \right]}_{D}^{24}$ −48.2 (*c* 0.05, MeOH) [41].

*(7S,8S)-Cilicione b* (**16**). Yellow solid. ${\left[\alpha \right]}_{D}^{24}$ +16.0 (*c* 0.1, MeOH) [42].

*(2S)-1,2-Butanediol* (**17**). Light yellow solid. ${\left[\alpha \right]}_{D}^{24}$ +7.8 (*c* 0.1, EtOH) [29].

### 3.4. Cell Culture

A549 (human non-small cell lung carcinoma cell), HCT116 (human colorectal carcinoma cell), SNU423 (human hepatocellular carcinoma cell), MCF7 (human breast adenocarcinoma cell), SH-SY5Y (human neuroblastoma cell), and HeLa (human cervical adenocarcinoma cell) were obtained from the Korean Cell Line Bank (Seoul, Korea). A549, HCT116, SNU423, and HeLa cells were maintained in the RPMI-1640 medium containing 10% FBS, 1% penicillin/streptomycin at 37 °C under 5% CO_2_ conditions. MCF7 and SH-SY5Y cells were cultured in the DMEM supplemented with 10% FBS and 1% penicillin/streptomycin at 37 °C under 5% CO_2_ conditions.

### 3.5. Cell Viability Assay

Cells were cultured on 96-well plates at a density of 5 × 10^4^ cells/mL and treated with compounds at the indicated concentrations. After 24 h of incubation, cell viability was analyzed according to the manufacturer’s instructions using the EZ-CyTox Cell Viability assay kit. Briefly, 10 μL of kit solution was added to each well for additional 4 h incubation. Absorbance was detected at 450 nm using a VERSA max microplate reader (Molecular Devices, Sunnyvale, CA, USA) and used to calculate the percentage of viable cells compared to the untreated cells. Results were expressed as cell viability (%) = (mean absorbency in test wells/mean absorbency in control wells) × 100. Cytotoxicity was expressed as the concentration of inhibiting cell growth by 50% (IC_50_ value).

### 3.6. Colony Formation Assay

Cells were treated with compounds **1** and **2** at indicated concentrations for two weeks. The medium was changed every three days by the treatment with compounds **1** and **2**. After that, the supernatant was thrown away, and the cells were washed three times with phosphate-buffered saline (PBS). The cells were fixed with 4% formaldehyde for 30 min and stained with 0.1% crystal violet for 30 min. Colonies were photographed, and the number of colonies was counted using Image J (National Institutes of Health, Bethesda, MD, USA) from three independent experiments.

### 3.7. Flow Cytometry Analysis

Annexin V positive MCF7 cells were detected using an EzWay Annexin V-FITC apoptosis detection kit according to the manufacturer’s protocol. Briefly, MCF7 cells were seeded and incubated with the indicated concentration of compounds **1** and **2** for 24 h. The cells were harvested, washed three times with PBS, and incubated with 1 × Binding Buffer. Then, the cells were incubated with 1.25 μL of Annexin V-FITC and 10 μL of propidium iodide (PI) at room temperature for 15 min in the dark. The samples were analyzed using a FACScan flow cytometer (Becton Dickinson, San Jose, CA, USA). The apoptosis percentage was calculated as the number of PI positive and Annexin-V positive cells divided by the total number of cells. The experiments were repeated three times independently.

### 3.8. Western Blotting

Cells were washed with PBS and lysed in lysis buffer (20 mM HEPES, pH 7.5, 150 mM NaCl, 10% glycerol, 50 mM EDTA, 1% Triton X-100) containing a protease inhibitor cocktail at 4 °C for 20 min. After centrifugation at 12,000 rpm for 15 min, the supernatants were collected. Protein concentration was determined using the Bradford protein assay (Bio-Rad, Hercules, CA, USA). Equal amounts of proteins were subjected to a 12% sodium dodecyl sulfate–polyacrylamide gel electrophoresis (SDS–PAGE) and transferred to a nitrocellulose membrane. The membranes were then blocked with 5% skim milk for 1 h and incubated with primary antibody. After washing, the membranes were incubated with a horseradish peroxidase (HRP)-conjugated secondary antibody for 1 h. Proteins bands were visualized using an enhanced chemiluminescence (ECL) system.

### 3.9. Statistical Analysis

All the data are presented as the mean ± SD and are representative of at least three independent experiments. Comparisons between the two groups were analyzed by Student’s *t*-test. A *p* value of less than 0.05 was considered to be statistically significant.

## 4. Conclusions

The leaves and (or) twigs of *J. sinensis* have usually been subjected to phytochemical and biological studies previously; however, this is the first report of the phytochemical study on the bark of this plant. In this study, bioassay-guided fractionation of an ethyl acetate-soluble fraction of the bark of *J. sinensis* using the A549 cell line, led to the isolation of 17 phenolic compounds **1**–**17**, which were found in this plant for the first time. Moreover, **14** and **16** have never been isolated from the family Juglandaceae. This study suggests that compounds **1** and **2** are the main compounds responsible for the biological activity for the bark extract of *J. sinensis*. The most active compound **1** is a potential candidate for antitumor drug based on its effective cytotoxic and apoptotic activities.

## Supplementary Materials

Supplementary materials can be accessed at: http://www.mdpi.com/1420-3049/21/1/120/s1.
