# Supplementary material for: Cytotoxic Compounds from Juglans sinensis Dode Display Anti-Proliferative Activity by Inducing Apoptosis in Human Cancer Cells
Source: Molecules. 2016 Jan 21;21(1):120. doi: 10.3390/molecules21010120 (PMC6274522; doi:10.3390/molecules21010120)
Supplement: Supplementary file 1 [file molecules-21-00120-s001.pdf]

Supplementary Materials: Cytotoxic Compounds from *Juglans sinensis* Dode Display Anti-Proliferative Activity by Inducing Apoptosis in Human Cancer Cells

Yoo Jin Lee, Jun Cui, Jun Lee, Ah-Reum Han, Eun Byul Lee, Ho Hee Jang and Eun Kyoung Seo

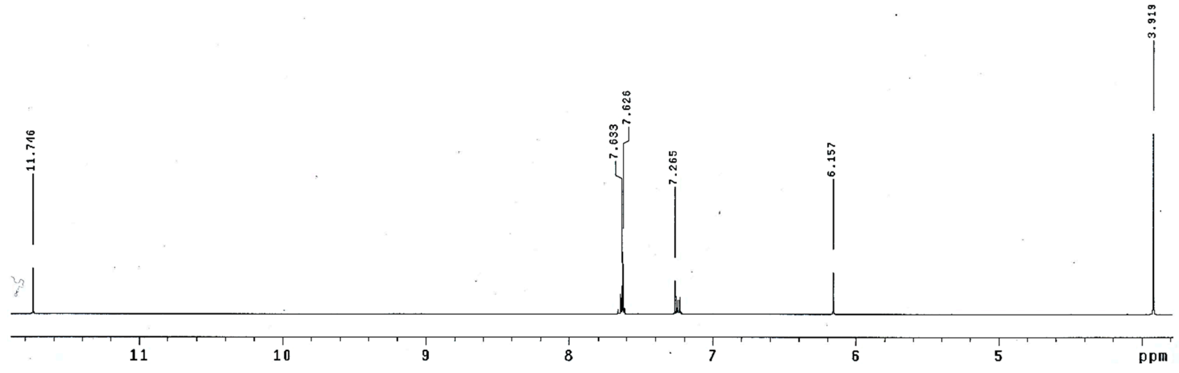

Figure S1. ^{1}H-NMR (400 MHz, $CDCl_3$) spectrum of compound **1**.

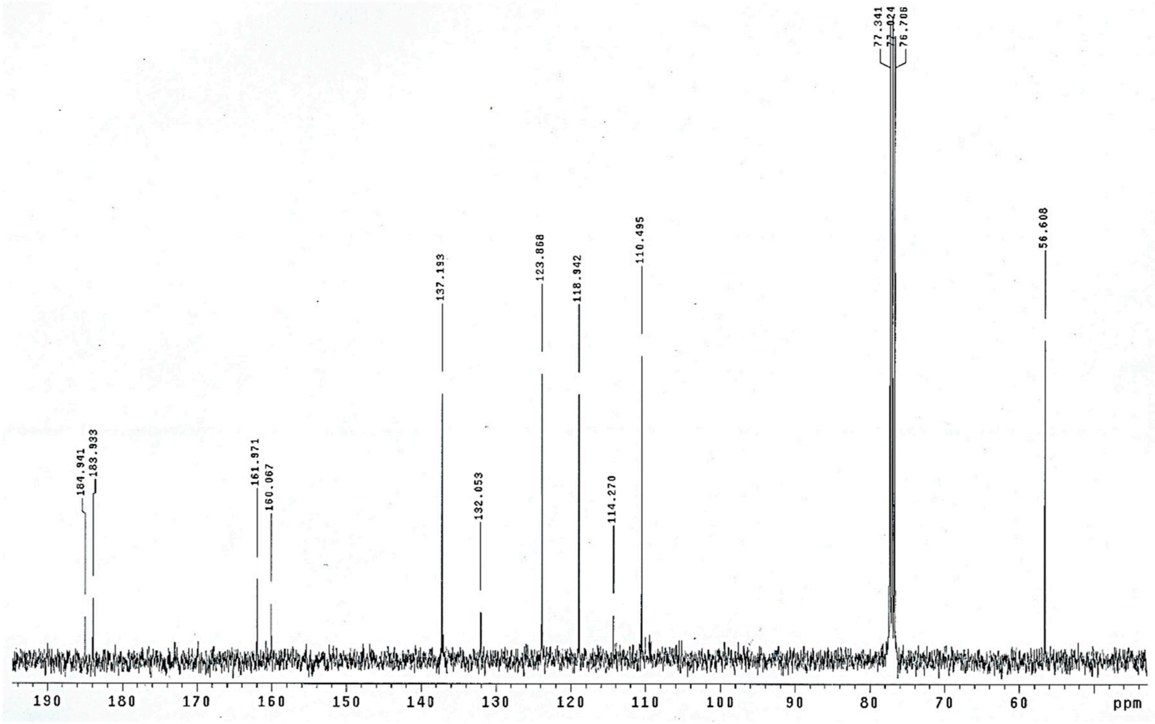

Figure S2. ^{13}C-NMR (100 MHz, $CDCl_3$) spectrum of compound **1**.

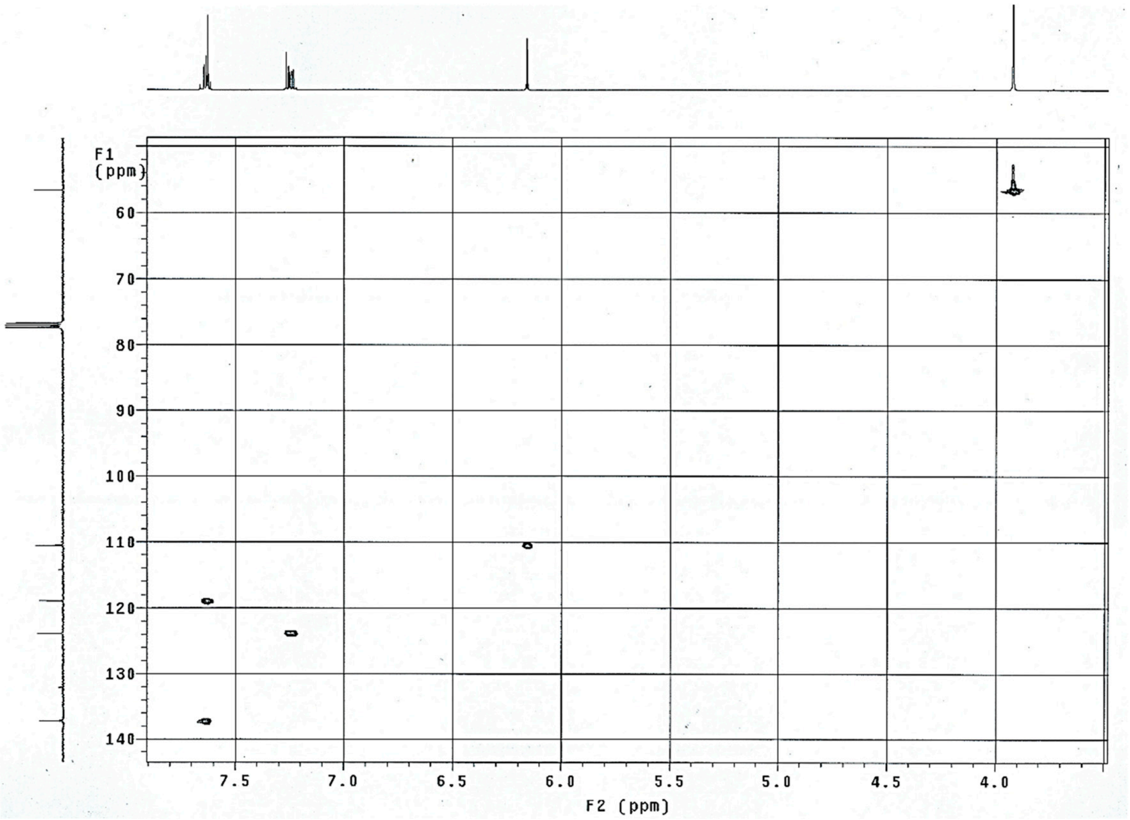

Figure S3. HSQC NMR spectrum of compound **1**.

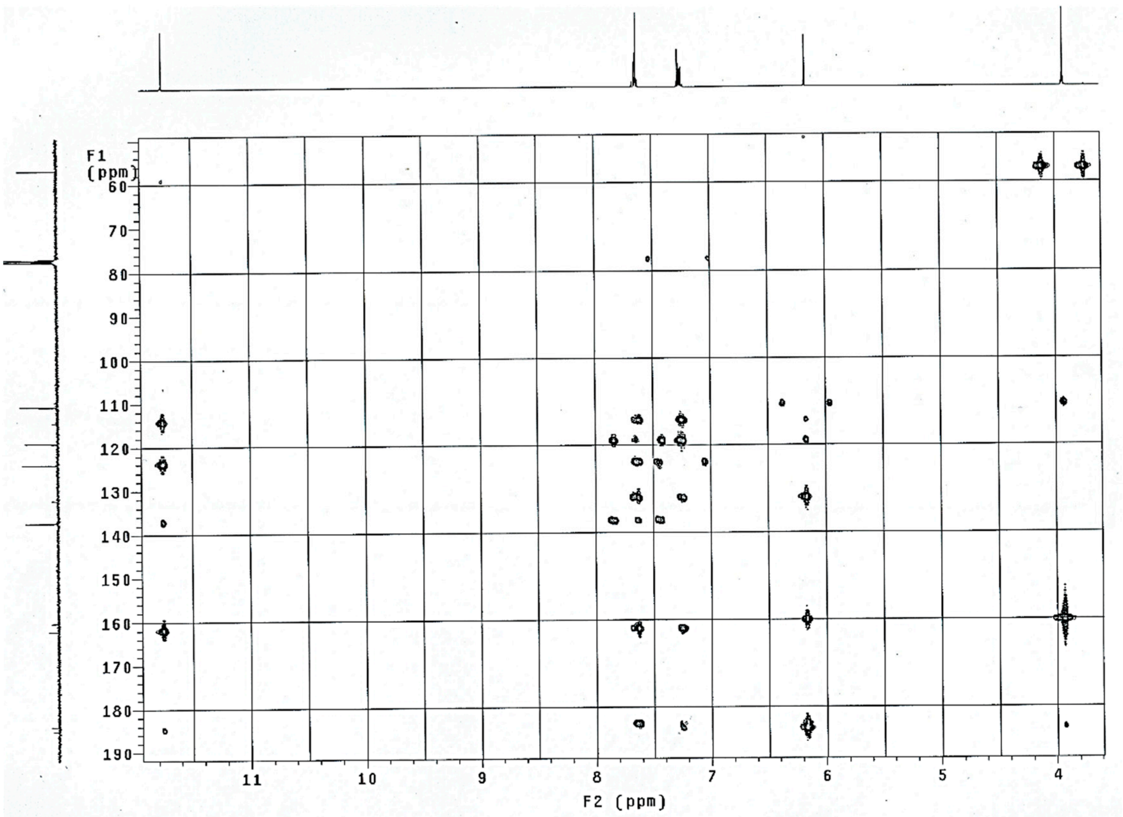

Figure S4. HMBC NMR spectrum of compound **1**.

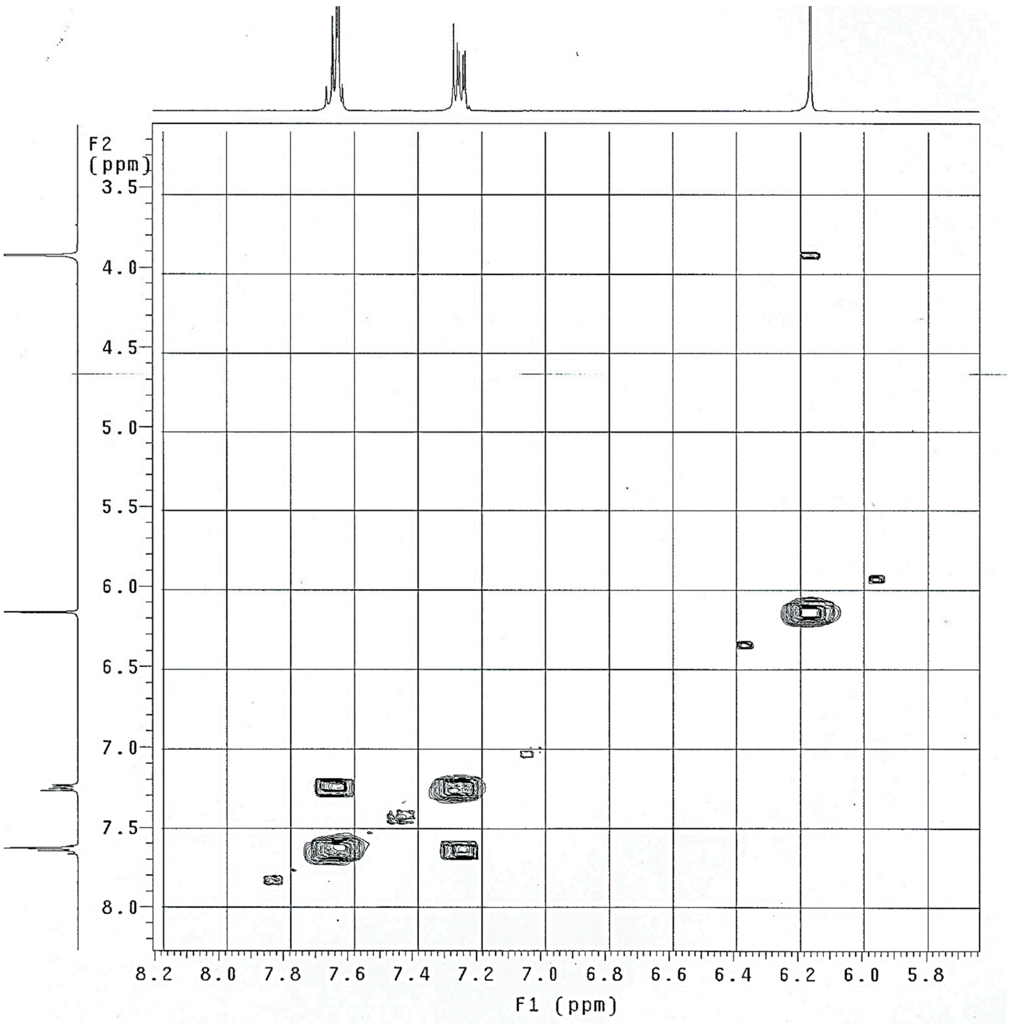

Figure S5. COSY NMR spectrum of compound **1**.

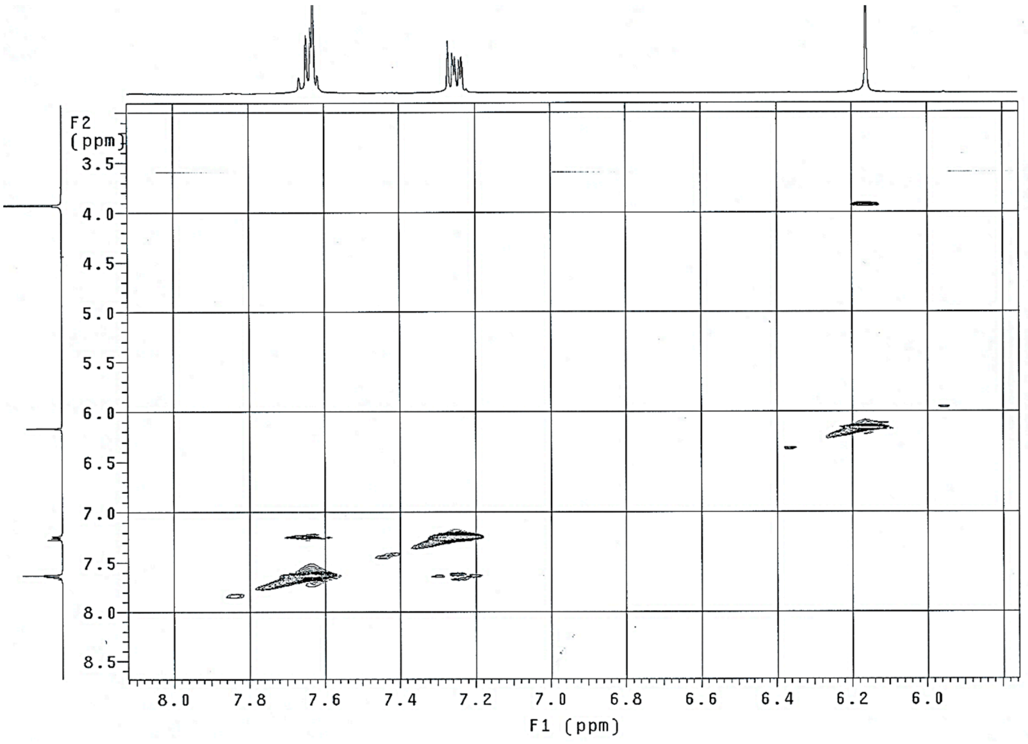

Figure S6. NOESY NMR spectrum of compound **1**.

NMR spectra for 5-hydroxy-2-methoxy-1,4-naphthoquinone (**2**)

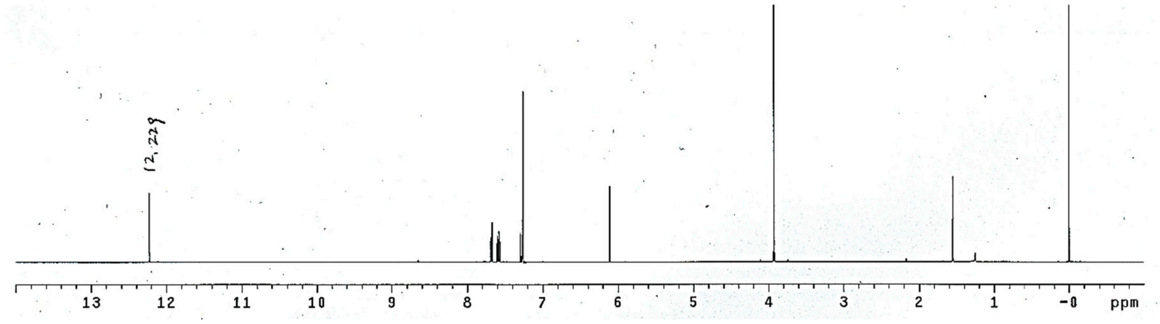

Figure S7. ^{1}H-NMR (400 MHz, $CDCl_3$) spectrum of compound **2**.

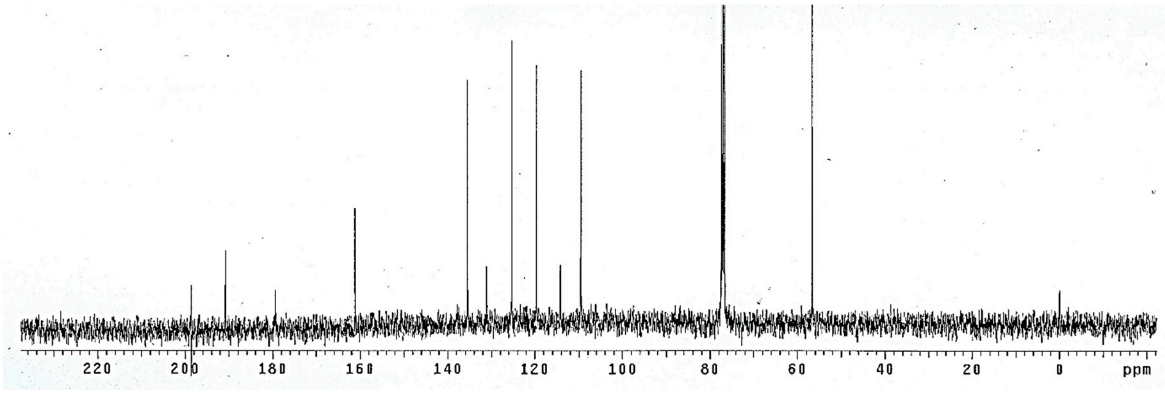

Figure S8. ^{13}C-NMR (100 MHz, $CDCl_3$) spectrum of compound **2**.

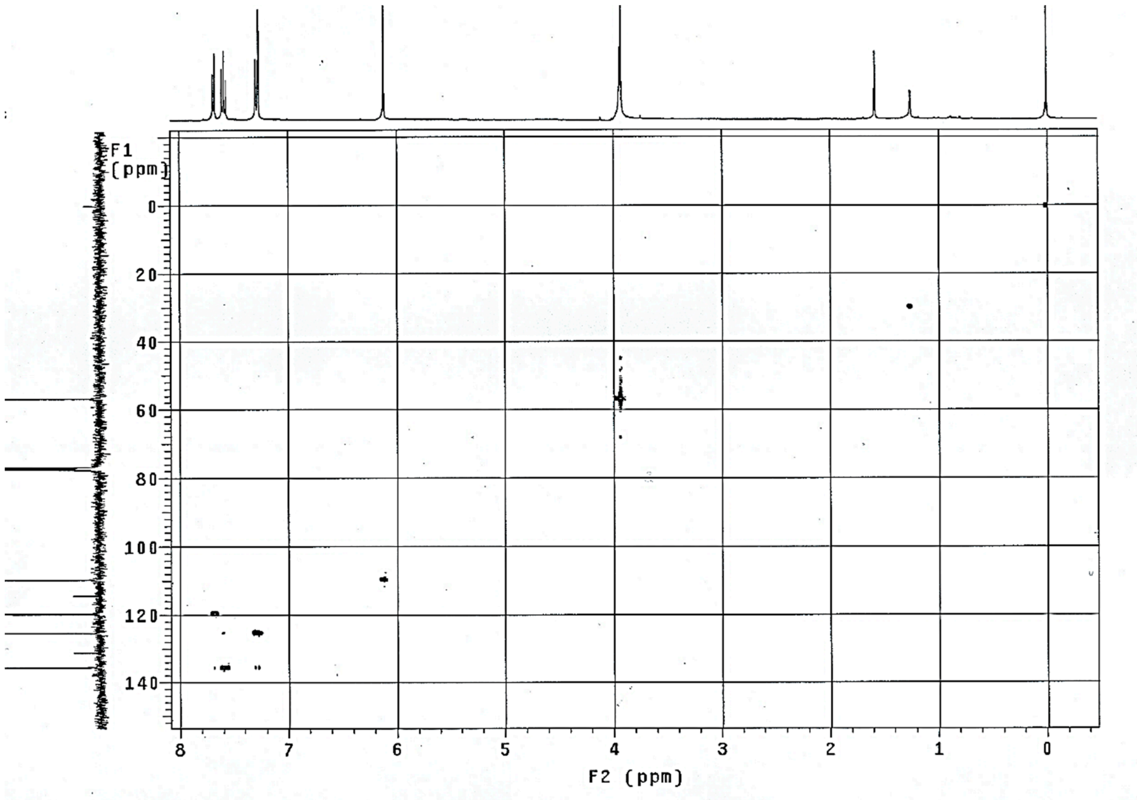

Figure S9. HSQC NMR spectrum of compound **2**.

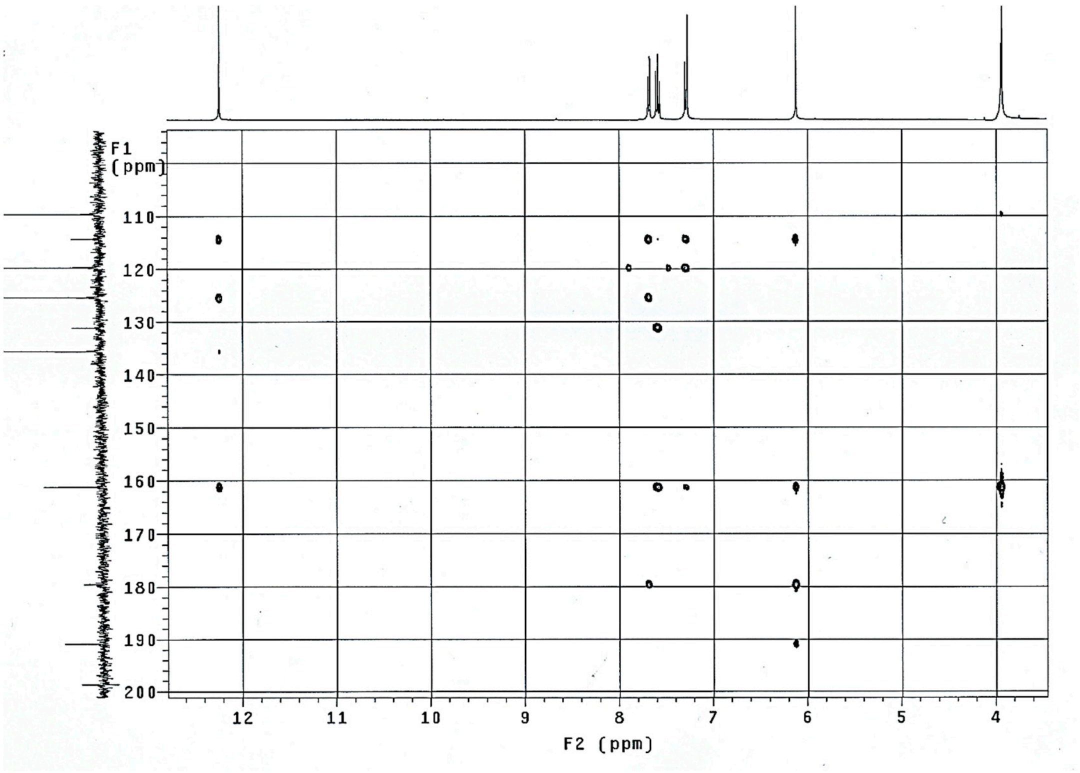

Figure S10. HMBC NMR spectrum of compound **2**.

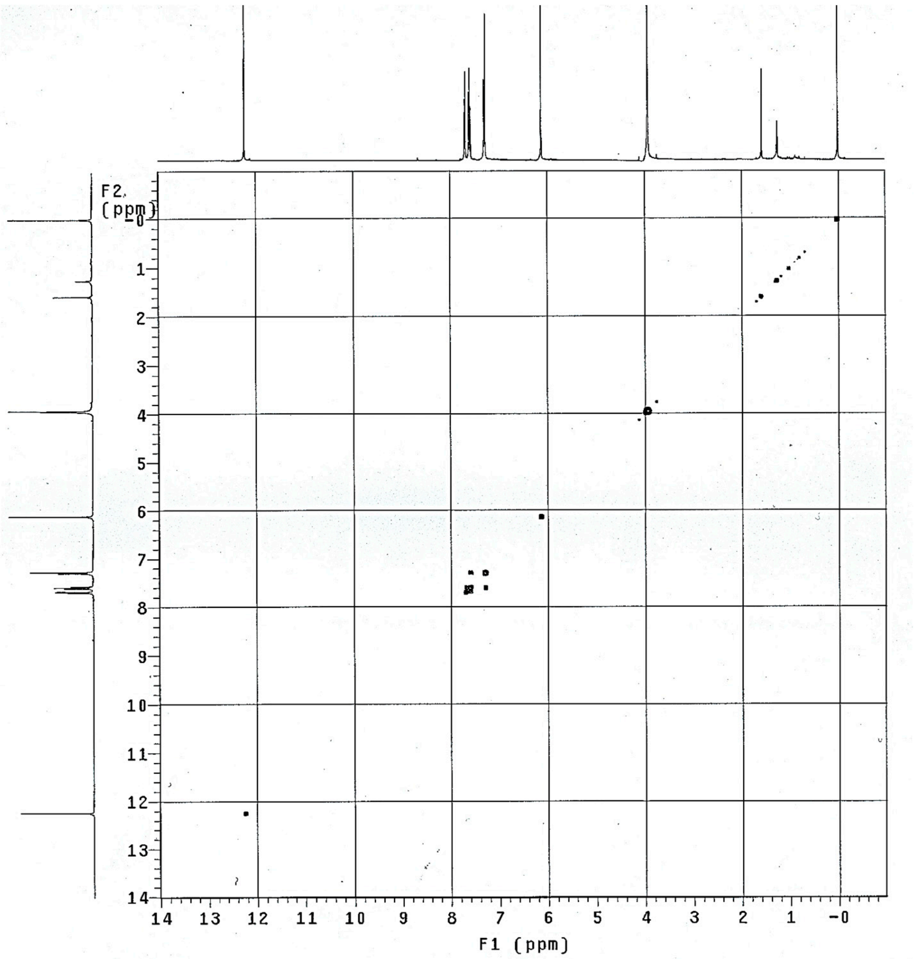

Figure S11. COSY NMR spectrum of compound **2**.

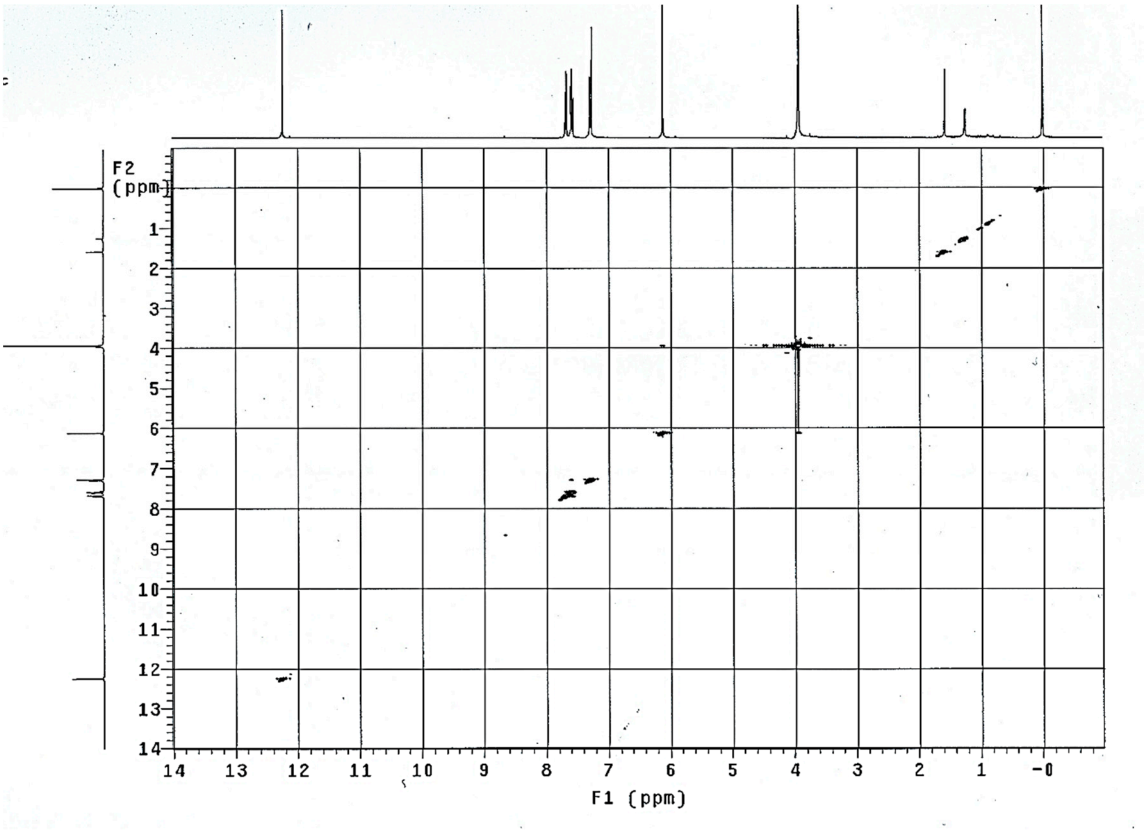

Figure S12. NOESY NMR spectrum of compound **2**.
